# Moderate temperature deposition of RF magnetron sputtered SnO_2_-based electron transporting layer for triple cation perovskite solar cells

**DOI:** 10.1038/s41598-023-35651-1

**Published:** 2023-06-05

**Authors:** Y. Zakaria, B. Aïssa, T. Fix, S. Ahzi, S. Mansour, A. Slaoui

**Affiliations:** 1 Qatar Environment and Energy Research Institute (QEERI), Hamad Bin Khalifa University (HBKU), Qatar Foundation, P.O. Box 34110, Doha, Qatar; 2 Laboratoire ICube‑CNRS, Université de Strasbourg, 67037 Strasbourg, France

**Keywords:** Energy science and technology, Materials science, Nanoscience and technology, Optics and photonics, Physics

## Abstract

The perovskite solar cells (PSCs) are still facing the two main challenges of stability and scalability to meet the requirements for their potential commercialization. Therefore, developing a uniform, efficient, high quality and cost-effective electron transport layer (ETL) thin film to achieve a stable PSC is one of the key factors to address these main issues. Magnetron sputtering deposition has been widely used for its high quality thin film deposition as well as its ability to deposit films uniformly on large area at the industrial scale. In this work, we report on the composition, structural, chemical state, and electronic properties of moderate temperature radio frequency (RF) sputtered SnO_2_. Ar and O_2_ are employed as plasma-sputtering and reactive gases, respectively. We demonstrate the possibility to grow a high quality and stable SnO_2_ thin films with high transport properties by reactive RF magnetron sputtering. Our findings show that PSC devices based on the sputtered SnO_2_ ETL have reached a power conversion efficiency up to 17.10% and an average operational lifetime over 200 h. These uniform sputtered SnO_2_ thin films with improved characteristics are promising for large photovoltaic modules and advanced optoelectronic devices**.**

## Introduction

The performance and cost-effectiveness fabrication of the perovskite solar cells (PSCs) are the two main assets which are increasingly attracting academic and industrial attention. Certified Power Conversion Efficiency (PCE) for the best solar cell efficiency has shown a 25.7% for PSCs as achieved by UNIST^1^. Focus is put nowadays on the PSCs commercialization^2^, and this aim is still facing two main challenges, namely a descent device operational-stability and the fabrication scalability. The stability of the PSCs has been the cornerstone of extensive research and development over the last years. Nevertheless, this research effort has been found to be one of the most complex physico-chemical issues that involves multiple factors and various physical phenomena. These issues are also a subject of the device configuration and materials’ characteristics. In fact, the device stability can directly be affected by the electrode material and its characteristics (work function, dimensions, etc.)^3^, electron transport layer (ETL) and hole transport layer (HTL) properties^4,5^, the nature of the interface between the absorber-perovskite layer and the charge transport materials^6^, and indeed, the stability of the perovskite material itself^7^. In 2016, Ahn et al.^8^ proposed that the ETL based on TiO_2_ is among the most responsible factors for the light-induced degradation in PSCs. This suggestion was also supported by the research outcome of Qiu et al.^9^. On the other hand, SnO_2_ as ETL has demonstrated its capability to replace the conventional TiO_2_ due to the fact that a PCE of more than 21% has been already achieved using SnO_2_ ETL^10^. SnO_2_ shows several benefits over TiO_2_, including a higher electron mobility and an excellent energy level matching^11^. More importantly, SnO_2_ as ETL is highly efficient against the perovskite solar cells degradation, which is induced by TiO_2_ ETL, thereby considerably improving the device operational lifetime under continuous light illumination at the maximum power point. In this context, Christians et al.^12^ have recently demonstrated a much longer lifetime with un-encapsulated perovskite solar cells based on SnO_2_ as ETL compared to TiO_2_. On the other hand, the second big challenge deals with the scalability of the PSC fabrication, to reach the module scale (i.e. perovskite solar modules (PSMs)), while maintaining performance similar to PSCs of small areas^2^. As large-scale thin film growth processes for the PSCs fabrication have been introduced, the number of reports related to PSMs has drastically increased^13^. For instance, Green et al. have reported a PCE of 16% with an aperture area (AA) of 16.29 cm^2^^14^ and Chen et al. have achieved a certified PCE of 12.1% with a larger AA of 36.1 cm^2^^15^. Other key parameters are related to the cost-effectiveness and large-scale deposition processes of ETL^2^. Currently, the majority of PSMs are based on TiO_2_ as ETL, which requires high processing temperature. TiO_2_ is also the origin of many instability issues^16^ due to its relatively higher resistance and a costly laser patterning method which is often used to remove TiO_2_ coating from the interconnection paths between sub-cells^17^. This is required to avoid the rise in the series resistance value, thereby decreasing the overall PSM performance^18^.

Unlike TiO_2_ material, SnO_2_ can be processed at much lower temperatures using different deposition technologies, including solution processes^11,19,20^, electrodeposition^21^, electron-beam^22^, atomic layer deposition^23^ and magnetron sputtering^24^. It is worth noting that the majority of reports so far related to SnO_2_ as ETL are only for small area devices^16^. Among all these thin fabrication methods, magnetron sputtering (MS) is one of the most promising technologies due to its advantages related to cost-effectiveness and uniform large-scale SnO_2_ thin films. Thus far, there are only a few reports about SnO_2_ thin films, deposited by MS, as ETL for PSCs. For instance, Ali et al. have reported a PCE of 14% for an area of 0.09 cm^2^^25^. Furthermore, the film uniformity across large area MS is demonstrated as well as the superior electrical conductivity and electron mobility of SnO_2_ versus that of TiO_2_ which are also demonstrated as an advantage to improve the interconnection quality between different sub-cells in PSMs. On the other hand, the state-of-the-art related to PCE of PSC based on SnO_2_ ETL is achieved by a very thin layer (~ 25 nm) of SnO_2_ deposited by spin coating technique^26^. However, this deposition method associated with the very thin layer can lead to fringe effects, pinholes, and thickness non-uniformity related to large area thin films, especially for upscaling small devices into large photovoltaic (PV) modules.

**Figure 1:**
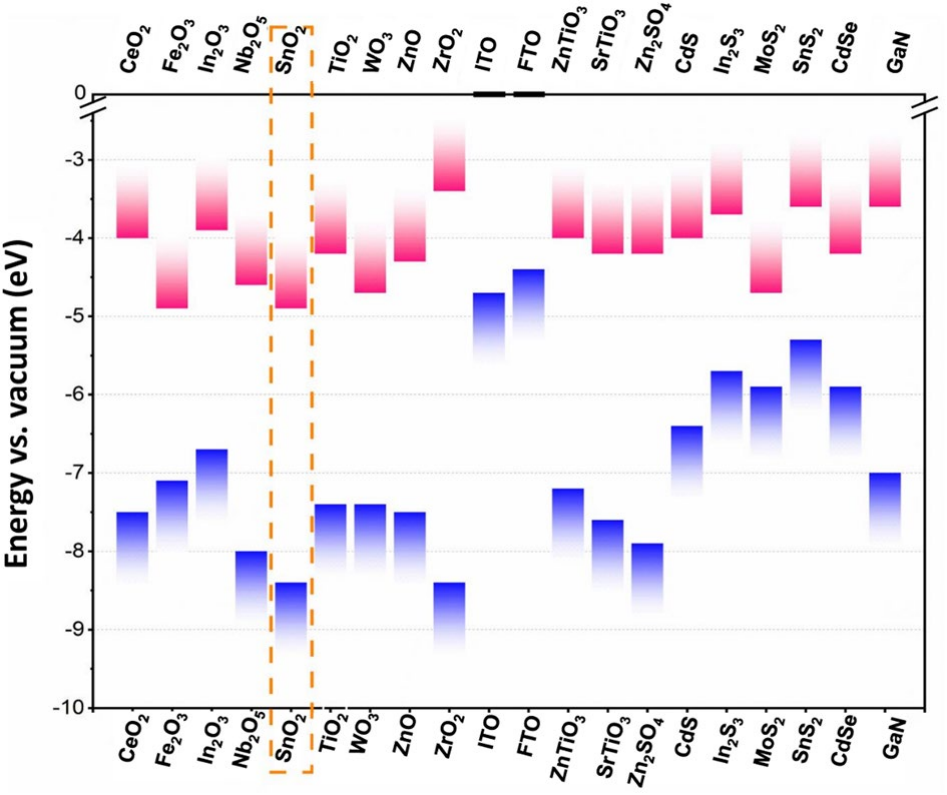
Schematic drawing showing the conduction band minimum (CBM) and the valence band maximum (VBM) of commonly employed inorganic metal materials as ETLs in PSCs^29–39^.

Figure 1 displays the conduction band minimum (CBM) and valence band maximum (VBM) of commonly implemented inorganic materials as ETLs in PSCs where metal oxides, metal sulfide, CdSe and GaN were included. To deliver an efficient and reliable PSC, it is a essential to meet the following key characteristics: (1) good optical transmittance; (2) a low photon-energy loss; (3) an appropriate bandgap-matching/alignment; (4) high electrical conductivity and electron mobility; (5) cost-effectiveness; and an acceptable rate of reproducibility (i.e. stability)^27,28^.

As a replacement to TiO_2_, various materials, which offer particular optoelectronic properties, have already been studied. This has included binary metal oxides (ZnO, In_2_O_3_, Nb_2_O_5_, WO_3_, Fe_2_O_3_ and CeO_2_)^32^, ternary metal oxides (Zn_2_SnO_4_, BaSnO_3_ and SrTiO_4_)^30^ metal sulfides (MoS_2_, CdS, In_2_S_3_, SnS_2_ and Bi_2_S_3_)^29^ as well as GaN, CdSe, and InGaZnO_4_^31^. Nevertheless, many pending issues are still to be addressed, including the low interfacial contact quality.

In 2015, the innovating work of Dai’s group demonstrated -for the first time- a PSC based SnO_2_ ETL with a PCE of 6.5%. This was followed, in the same year, by the work of Wan et al. who reached a PCE of more than 15%^20,40^. The power conversion efficiency of PSC based on SnO_2_ as electron transport material has recently achieved values above 20%^10^. This has demonstrated the SnO_2_ as an attractive and promising ETL material from various point of views, especially for perovskite solar cell, and as an excellent substitute to the conventional TiO_2_, owing to the following factors: (1) an optimized energy-level matching^41^; (2) a significantly higher electron mobility than TiO_2_; (3) a high electrical conductivity^42,43^; (4) a large bandgap (3.6–4.5 eV); (5) a high transparency (i.e. optical transmittance)^44,45^; (6) a large flexibility in terms of processing temperature from relatively low temperature down to room temperature; (7) a high stability under light (i.e. low photoactivity), and (8) a high stability under heat and humidity^46,47^.

In addition, spin coating or spray methods are usually used for synthesis of TiO_2_ ETL in PSC. This requires an elevated post-treatment temperature, generally above 450 °C, to enable the formation of the mesoporous layer with a dense structure, a crystalline structure, and a good electrical conductivity. Conversely, SnO_2_ is routinely grown at much lower temperatures (≤ 250 °C) and in some cases it is grown at room temperature when the crystalline structure is not required. This advantage is very appealing for large-scale industrial applications.

In this work, we have achieved the implementation of moderate temperature radio frequency magnetron sputtered SnO_2_ as electron transport layer for spin-coated triple-cation based perovskite solar cells. We demonstrated that both procedures of mesoporous-scaffold and high-temperature processing are not essential in order to achieve high PSC device performance. Furthermore, no passivation process has been performed and no encapsulation has been used. Nevertheless, a PSC of more than 17% PCE has been demonstrated.

We have explored the material’s properties of SnO_2_, namely structural, morphological, electrical and optical properties, as well as its chemical states. We have also studied the temperature dependency of the perovskite layer performance through PL measurement under various temperature, which has suggested an interplay of different physical phenomena, including charge transfer dynamics and charge recombination. Our developed MS SnO_2_ ETL is demonstrated to achieve a good device PV performance and a relatively good lifetime, which also could help for further development and integration of SnO_2_ films into PSMs.

## Results and discussion

To prepare metal-oxide thin films with a high quality, either in the lab- and/or at an industrial-scale, magnetron sputtering is demonstrated to be a reliable and mature deposition process, also offering the possibility to use low-cost materials’ targets. The SnO material is sputtered through high-energy argon-plasma ions, it reacts with oxygen and then it is deposited on the top of fluorine-doped tin oxide (FTO) layer under a continuous process. An accurate control of the film thickness and density can be achieved with MS via the deposition rate. It’s a relatively cost-effective process, with very low rate of waste. The thin film growth process is usually operated in a high vacuum chamber, which enables a high level of reproducibility^48^.

Qiu et al. studied in a systematic way the physical and chemical properties of the sputtered SnO_2_^26^. The fabricated perovskite solar cells implementing SnO_2_ as ETL were found to exhibit a PCE of about 20% and a stability of about 625 h as measured under T80 standard, thereby demonstrating the enhanced electrical conductivity and stability thanks to SnO_2_^26^. On the other hand, Bai and co-workers highlighted the impact of the Ar/O_2_ gas ratio on the structural and morphological characteristics of the sputtered SnO_2_ films^49^. The level of the trap states and dynamics of the carrier transit were also investigated in their PSC devices, which demonstrated a champion PCE up to 18%^49^. Moreover, Otoufi et al. experimented the bilayer architecture made with sputtered SnO_2_ on TiO_2_ layer, which was found to improve charge collection capacity, which has led to a PCE of about 12%, which is 4% higher than that obtained with only TiO_2_ as ETL (~ 8%)^50^. It is worth noting at this level that the flowing gas during the deposition process plays a key role in controlling the oxygen vacancies^51^. In addition, defects states within the SnO_2_ bandgap, which may originate from the amorphous and/or nano-crystallinity present in the films, might be effectively suppressed by a post-thermal annealing process under air, thereby leading to the passivation of the interface with the perovskite material.

In order to study the material’s properties, two SnO_2_ thin film samples were prepared on glass substrate; (1) as-deposited SnO_2_, and (2) air-annealed SnO_2_ at 250 °C for 30 min.

**Figure 2:**
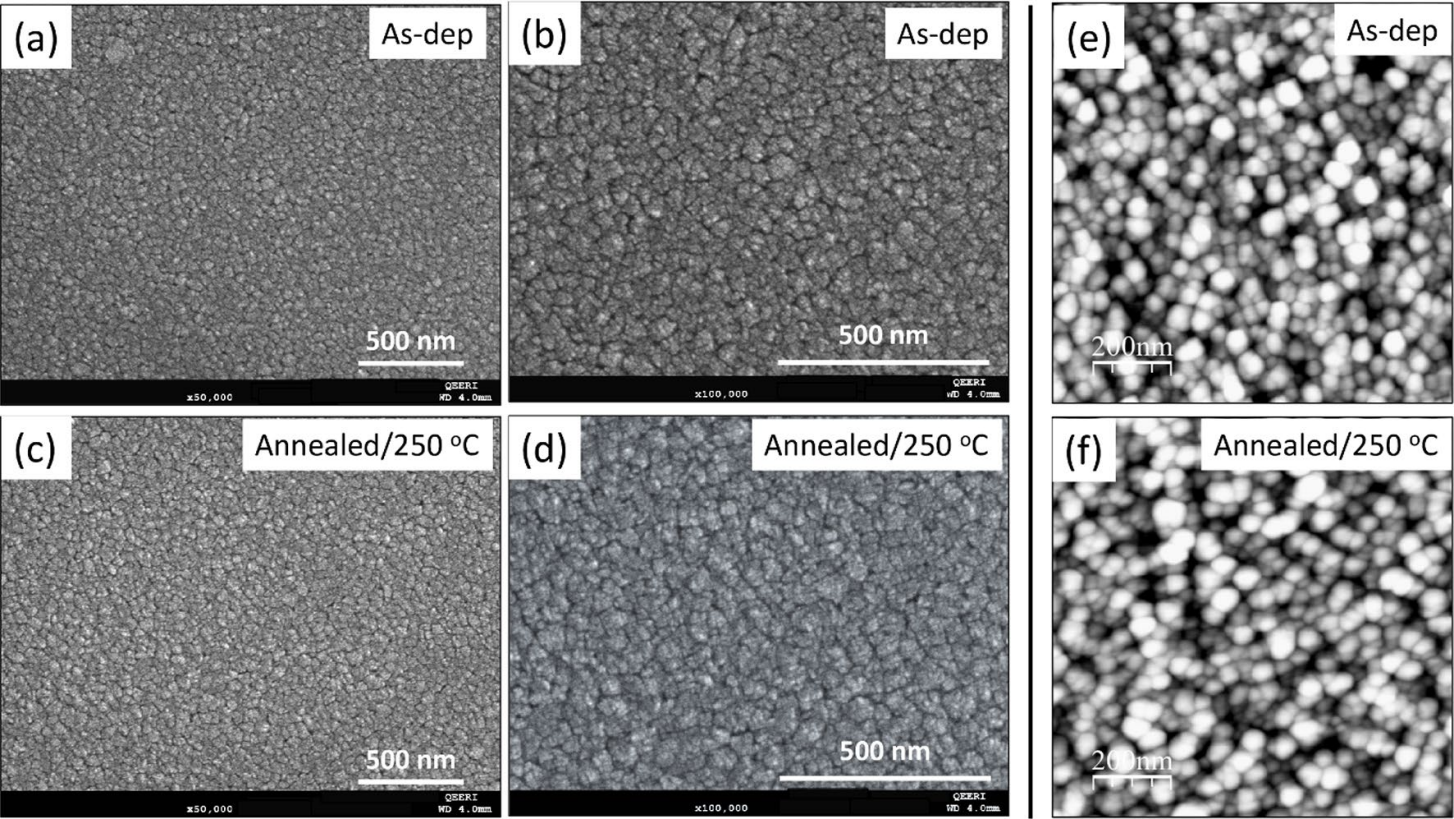
SEM (**a**–**d**) and AFM images (**e**, **f**): Top-view SEM images showing the SnO_2_ films (**a**) as deposited magnified at x50k, (**b**) as deposited magnified at x100k, (**c**) annealed at 250 °C magnified at x50k, (**d**) annealed at 250 °C magnified at x100k; and AFM images of the SnO_2_ films (**e**) as-deposited, (**f**) annealed at 250 °C.

Figure 2a–d show representative top-view SEM images of as-deposited and thermally annealed SnO_2_ films deposited onto glass substrate. SnO_2_ samples demonstrate a uniform smooth surface morphology, with a large grain size, pinhole- and crack-free films. The thermal annealing treatment was found to have little or no impact on the morphology of the SnO_2_ thin films. Figure 2e,f show the associated AFM images of these SnO_2_ films.

**Figure 3:**
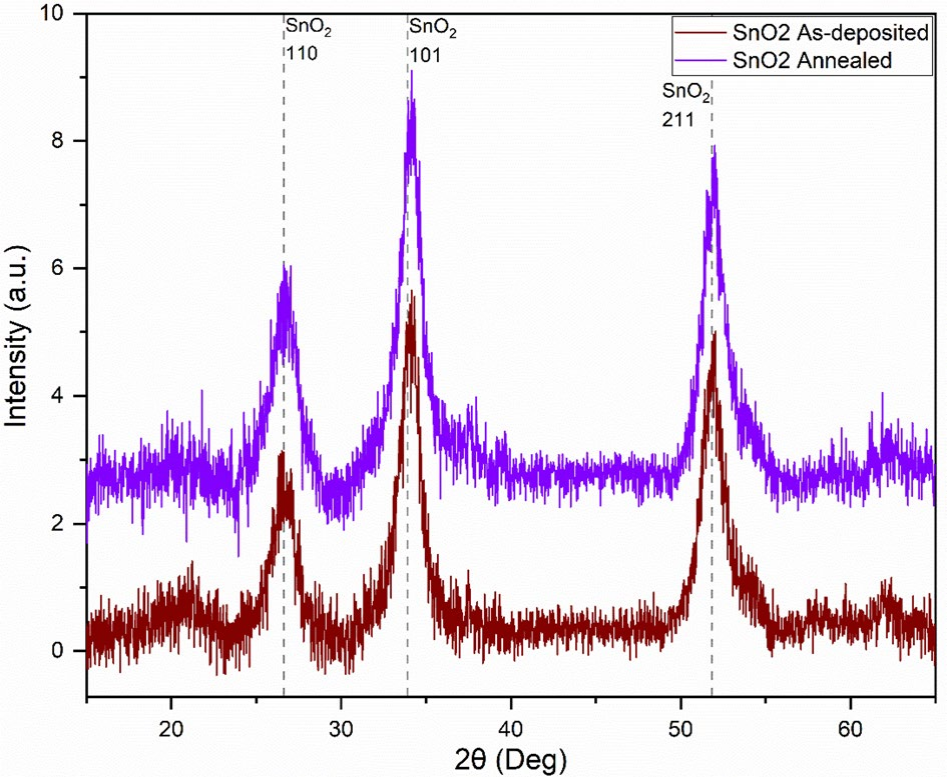
GIXRD for as-deposited SnO_2_ and annealed SnO_2_ thin film samples.

The as-deposited SnO_2_ thin films have shown a clear crystalline microstructure of the SnO_2_ phase by displaying clear triple peaks of (110), (101) and (211) orientation planes, as shown in Fig. 3. There is no presence of any secondary phases which reveals the high crystalline quality of this SnO_2_ thin film. After the air annealing the SnO_2_ thin film samples have kept their SnO_2_ microstructural phase as revealed by the same observed triple peaks of (110), (101) and (211) (Fig. 3). Furthermore, it is noticed that the crystallinity has enhanced after the air annealing as demonstrated by the slight decrease of the full width at half maximum (FWHM) related to the SnO_2_ phase peak (101). By using the Scherrer equation, the calculated crystallite size of SnO_2_ phase using (101) peak has increased from 60.7 Å for the as-deposited SnO_2_ sample to 69.2 Å for the annealed SnO_2_ sample. These results are shown in Table 1. As per the GIXRD analysis, it is clear that the growth conditions favored the formation of SnO_2_ phase without any secondary phases. Furthermore, the post air annealing process has kept and enhanced the crystallinity of the SnO_2_ phase without promoting any secondary phases. These results are matching with previously reported findings^51^ where improved crystallinity of the SnO_2_ films was demonstrated with respect to an air-thermal-annealing treatment, as more oxygen incorporated in the film increased its crystallinity.

**Table 1:** Crystallite size calculation using SnO_2_ phase peak (101) from Scherrer equation.

Sample	FWHM (°)	Crystallite size (Å)
SnO_2_ as deposited	1.36	60.7
SnO_2_ annealed	1.34	69.2

**Figure 4:**
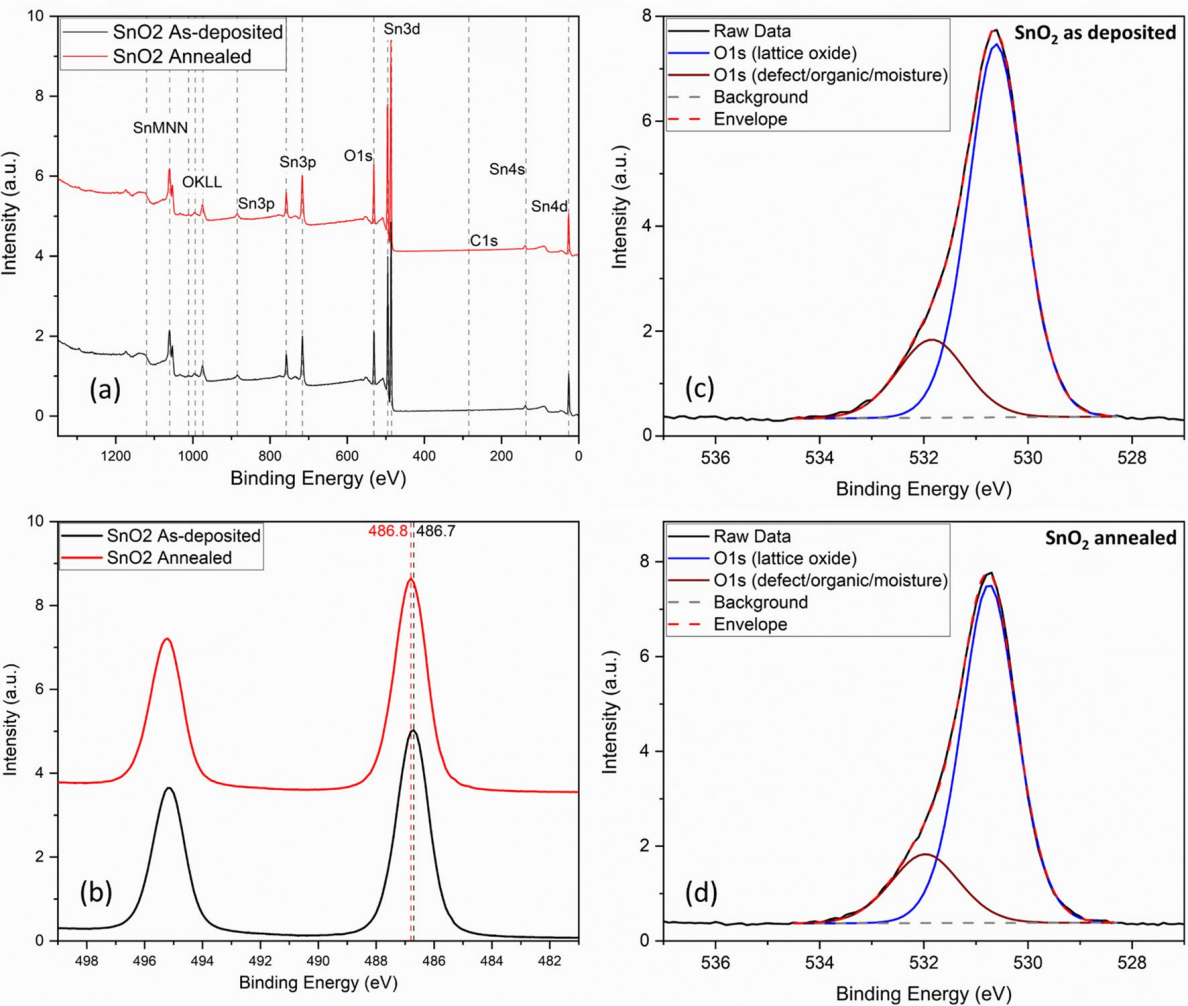
Post cleaned XPS spectra for as-deposited SnO_2_ and annealed SnO_2_ thin film samples: (**a**) surveys, (**b**) Sn3d, (**c**) O1s for as deposited SnO_2_, (**d**) O1s for annealed SnO_2_.

Survey XPS spectra has revealed the high purity of the SnO_2_ thin film samples owing to the presence of only O and Sn as well as the low-to-absent content of C, particularly after the surface cleaning. All the peaks are related to Sn and O photoelectrons and auger electrons as shown in Fig. 4a. For the as deposited SnO_2_ samples, Sn3d_5/2_ peak is positioned on a higher binding energy at 486.7 eV, which indicates the presence of higher oxidation state related to Sn(IV). After the air annealing process, the peak position of Sn3d_5/2_ has slightly shifted towards the higher binding energy at 486.8 eV, which indicates a slight increase in oxidation state after the annealing treatment as displayed by Fig. 4b. These results match well with the crystallite size increase as discussed above. XPS related O 1 s spectra have shown two main component peaks, the first peak is related to the photoelectrons originating from the oxygen atoms in the SnO_2_ lattice, while the second peak is related to the other oxygen chemical states which might be defects in Sn oxide, or remaining surface organic molecules, and/or surface adsorbed moisture, as displayed in Fig. 4c,d. For the as-deposited SnO_2_ sample, the peak position of O related to the lattice SnO_2_ is located at 530.6 eV, which confirms the presence of Sn(IV) oxide related to SnO_2_. This is also corroborating well with the previous GIXRD results. The lattice oxide peak FWHM is of 1.26 eV, which is a reduced value that indicates the low chemical disorder as expected to be achieved by the vacuum-based material deposition. After the annealing, the peak position of O related to the lattice oxide has increased to 530.8 eV, which reveals the higher oxidation state of SnO_2_ thin film after the annealing. Furthermore, the FWHM of O related to lattice oxide has slightly decreased from 1.26 to 1.25 eV, revealing a slight enhancement in the chemical disorder. The percentage of O related to lattice oxide has slightly increased from 79.8 to 80.1%, which also reveals that the thermal annealing process has improved the micro-structuring of the SnO_2_ films as shown in Table 2 and it has enabled a slight reduction of the oxygen vacancies. Overall, this process has improved the surface chemistry of the SnO_2_ thin film^52^.

**Table 2:** XPS O1s spectra fitting: peak positions, FWHM and percentage of chemical states.

Sample	Binding energy (eV)	FWHM (eV)	Chemical state	Percentage (%)
SnO_2_ as deposited	530.6	1.26	Lattice oxide	79.8
	531.9	1.54	Defect/organic/H_2_O	20.2
SnO_2_ annealed	530.8	1.25	Lattice oxide	80.1
	532.0	1.54	Defect/organic/H_2_O	19.9

**Figure 5:**
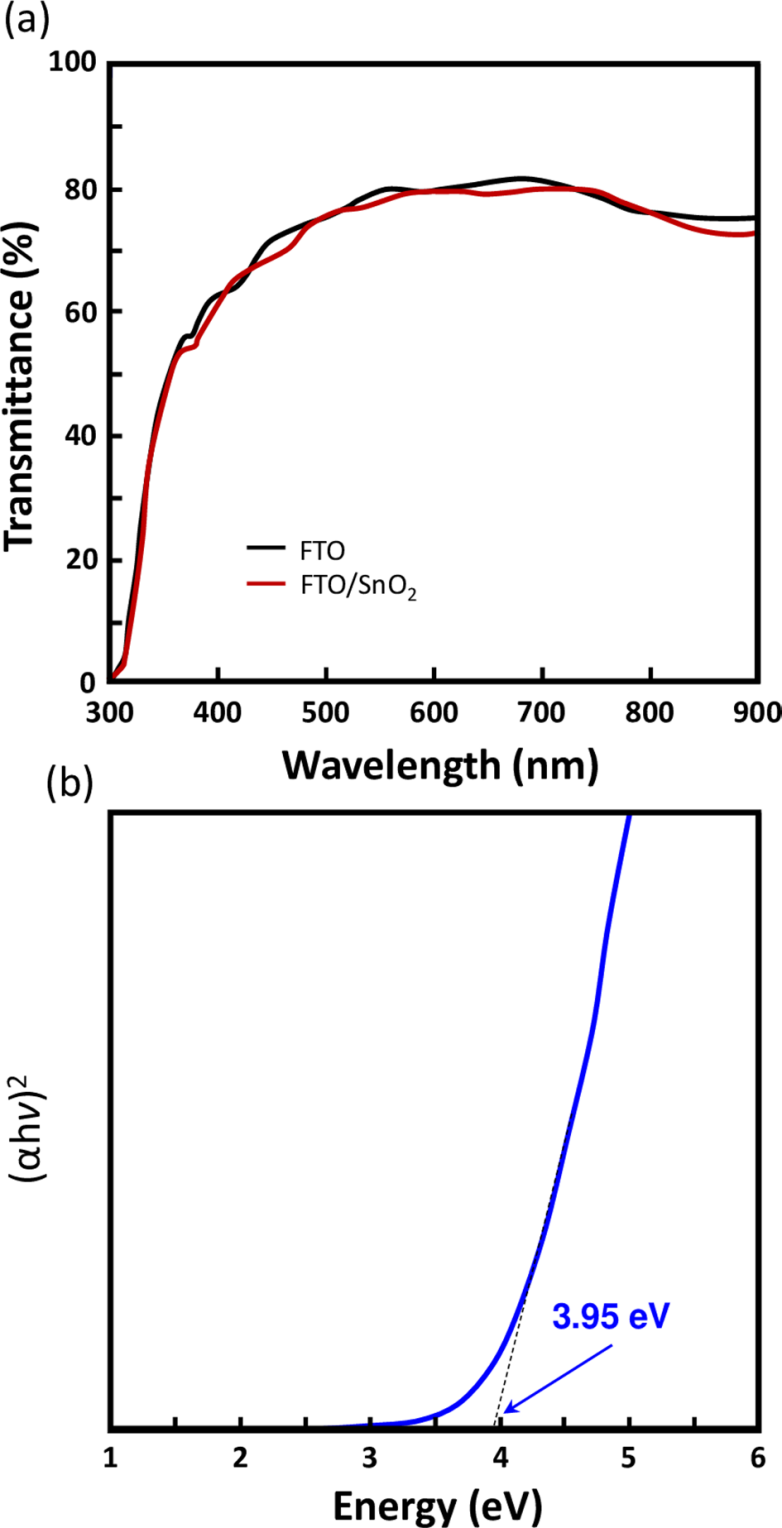
(**a**) Transmittance spectra % of the sputtered SnO_2_ film deposited on fluorine doped tin oxide (FTO) glass and FTO on glass only. (**b**) Associated Tauc plot showing a bandgap of 3.95 eV.

The SnO_2_ films have shown rather a high optical transmittance in the visible range which is higher than 80%. The associated bandgap obtained from Tauc plot was about 3.95 eV, a value that is greater than that of TiO_2_ and/or ZnO thin films (Fig. 5). It is worth noting that larger bandgap can act as an efficient hole blocking and as a barrier against high-energy photons absorption, thereby decreasing the current losses, which is a requirement for stable halide perovskite solar cells. Indeed, both high optical transmittance and film quality are critical for an effective ETL layer for the *n-i-p* planar structure of PSCs^53^.

Furthermore, Kam et al.^53^ used a semiconductor band structure to calculate the position of the conduction band minimum (CBM) of a sputtered SnO_2_ film, which was found to be − 4.36 eV, which is even lower than that of TiO_2_ and ZnO, where both are around − 4.2 eV. More specifically, a deeper conduction band value will improve the electron transfer from the absorber layer, i.e. perovskite film, to the SnO_2_ ETL layer. In the same context, Kam et al. calculations also showed the position of valance band maximum (VBM) at − 8.08 eV^53^, which is clearly deeper than that of TiO_2_ and ZnO, which are at − 7.4 eV and − 7.6 eV, respectively. Here again, a deeper valence band of SnO_2_ conjugated to its larger bandgap will enhance the capacity of the perovskite film to block the holes towards the SnO_2_ ETL.

On the other hand, the electrical resistivity of SnO_2_ films has decreased from 0.245 Ω cm (as-deposited) to 0.134 Ω cm after a thermal annealing in air. The associated electron mobility has increased from 4.38 cm^2^/V.s as-deposited to 11.29 cm^2^/V.s after annealing treatment. However, the electron density has slightly decreased from 5.82 × 10^18^ cm^−3^ to 3.86 × 10^18^ cm^−3^. The as-deposited and the annealed SnO_2_ samples have negative charge carrier type (electrons). The thermal annealing process has clearly enhanced the electrical conductivity by significantly increasing the electron mobility and this finding is matching with the increase in crystallite size after the annealing process. The decrease in charge carrier concentration is related to the oxygen vacancy reduction as the thermal annealing in presence of oxygen in air enables the vacant oxygen site filling. On the other hand, its effect on the surface morphology is not very evident. The effect of the thermal annealing has also been highlighted and discussed above through microstructural study and chemical state analysis as well as the measurements of the average roughness as conducted by AFM (Fig. 2e,f). In fact, the root mean square (RMS) roughness value was found to be in the low-level values, and it changed only slightly between the as-deposited and the annealed film (measured in the range of 1.45–1.33 nm). Moreover, from morphological point of view, the sputtered SnO_2_ film was uniformly deposited, thereby showing rather a low value of surface roughness. This process is also boosted by thermal annealing treatment, and the obtained roughness values are highly accommodating for the solution-deposited perovskite on SnO_2_/FTO/Glass. Therefore, this critical issue of the roughness effect has not been discussed in depth in the relevant literature. It is worth noting that a higher level of roughness of SnO_2_ film will lead to inconstant rate for perovskite crystallization and hence leads ultimately to an increase of the carrier recombination probability between the perovskite absorber layer and the SnO_2_ ETL.

**Figure 6:**
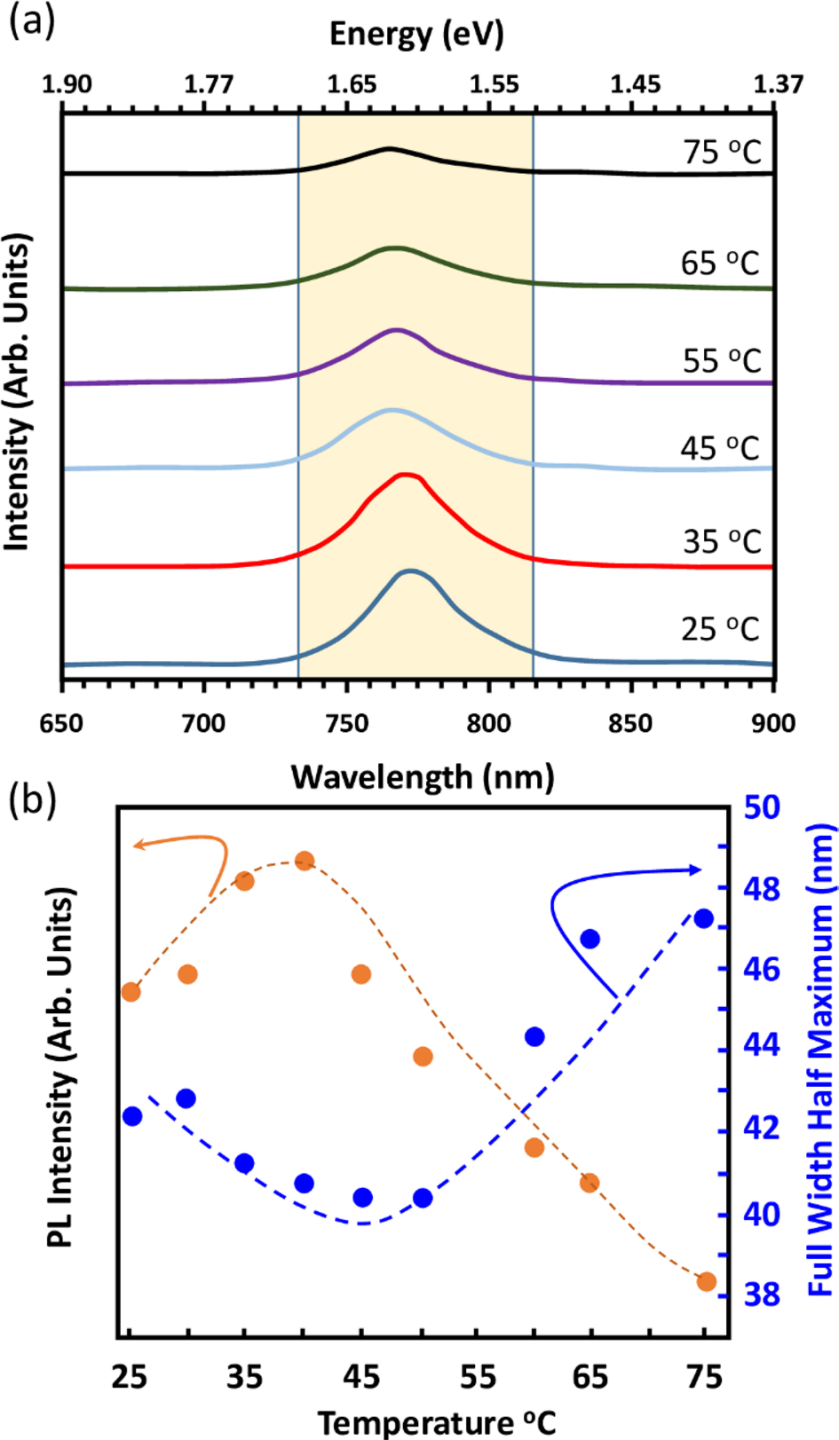
(**a**) Variation of the PL spectrum of the absorber perovskite films deposited on glass substrate. Measurements are performed in the range 25–75 °C. PL λ_ext_ = 532 nm. (**b**) Associated change in the PL peak intensity at ∼ 773 nm and the FWHM as a function of temperature.

To highlight the impact of real-world conditions on these PSC devices, especially when they are subject to operate in a harsh condition, such as desert environment, a temperature-sensitive Photoluminescence (PL) study was conducted through PL spectroscopy to elucidate the correlation with the charge-carrier and bandgap dynamics. Figure 6a shows the temperature-dependency of the PL measurements of the tri-cation perovskite films deposited on glass substrates. A well-defined PL peak centered at ∼ 773 nm is measured and is the fingerprint of the band-to-band recombination. It shows an associated bandgap of ∼ 1.6 eV, corresponding typically to the tetragonal phase of the tri-cation perovskite material^13^. The variation in PL emission peak position (Fig. 6a) as well as in PL emission peak broadening and intensity (Fig. 6b) as a function of temperature, were investigated and the results are presented in Fig. 6. The PL intensity was found to increase with respect to the temperature up to ∼ 40 °C and then start decreasing when the temperature continues increasing from 40 to 75 °C.

A rise in the PL emission intensity is generally the consequence of a decrease of the non-radiative recombination at traps and defects level in the bandgap, which lowers the band-to-band recombination rate and lower the charge–phonon coupling in the perovskite film^15,54^, leading thereby to an improvement of the V_oc_ and the fill factor (FF) values. Moreover, this decrease in the PL-emission-peak intensity while increasing the temperature above 40 °C is because of the charge trapping due to a boost of the charge–phonon coupling. This is also accompanied by a PL-emission-peak broadening (Fig. 6a)^13^. The variations in the charge–phonon coupling and the orbital splitting, which are a direct consequence of the perovskite lattice expansion with respect to the temperature increasing, generate a singular bandgap broadening in addition to a blue shift of ∼ 15 meV in the PL emission^13^. At this range of temperature, both the frequency and the population of the particular involved phonon modes increase with temperature^54^ and support the suggestion that the charge trapping due to the electron–phonon coupling is dominant, which might be the reason behind the reduced PSC performance at elevated temperatures. Figure 6b shows an additional highlight of the temperature-dependency of the charge–phonon coupling^13,55^, namely the variation of the FWHM of the PL emission peak with respect to the temperature. The FWHM was found to decrease slightly from RT to 40 °C and then noticeably increase from 40 to 75 °C, denoting a higher charge–phonon interaction at this range of temperatures. This broadening of the FWHM further supports the diagnosis and conclusion that PSC PV performance decrease for temperatures beyond 40 °C could be attributed to the charge trapping related to higher charge–phonon interactions. Overall, we attribute the PL changes up to 40 °C to the carrier accumulation near the perovskite/glass interface and/or to the diminution in the non-radiative charge traps, whereas higher charge–phonon interactions dominate at higher temperatures.

**Figure 7:**
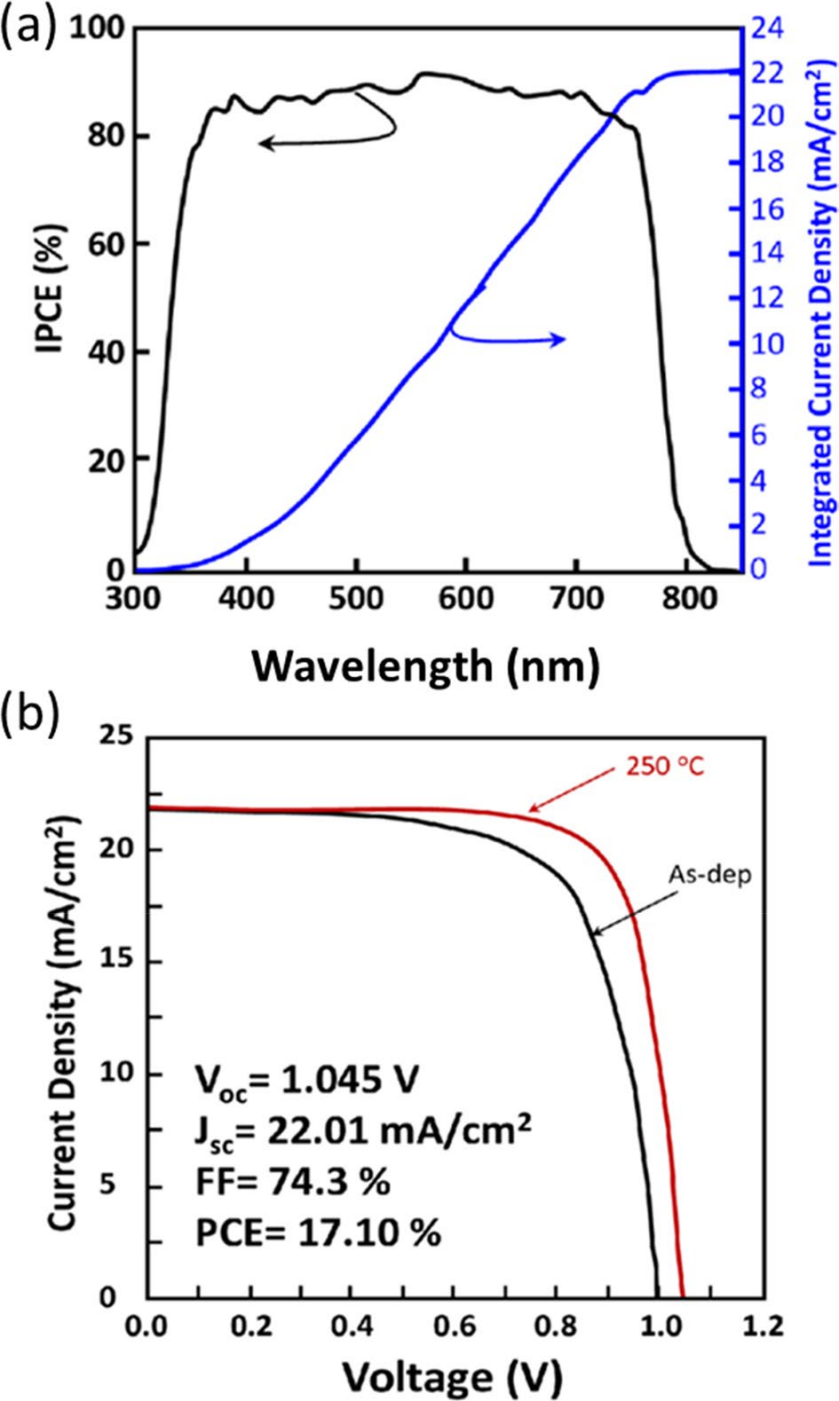
Perovskite solar cell device performance. (**a**) IPCE and associated integrated current density of the best device based on the sputtered SnO_2_ films as ETL. (**b**) *J–V* curves of the perovskite devices based on the SnO_2_ films as-deposited and that annealed at 250 °C (device performance results are related to the annealed SnO_2_ at 250 °C).

Figure 7 displays the perovskite planar-junction solar-cell performance of the best device based on the optimized sputtered SnO_2_ films integrated as ETL. Figure 7a shows the incident-photon-to-current efficiency (IPCE) and associated with the integrated current density. Following the optimization process of SnO_2_ thin films using the growth and post-depositions conditions, an optimized SnO_2_ ETL could be achieved through improving its material properties related to micro-structure, morphology and surface chemistry as well as the optoelectronic properties. Therefore, a PCE of 17.1% has been reached as shown in Fig. 7b after the post-annealing treatment. The improvement of PCE from as-deposited SnO_2_ ETL of 15.07% to thermally annealed SnO_2_ ETL of 17.1% is a direct result of the improved microstructural and optoelectronic properties, including electrical conductivity and electron mobility. As discussed above, SnO_2_ has high transmittance in visible light region and a deep valence band maximum position which has improved the hole-blocking process while minimizing the recombination at the SnO_2_/perovskite interface. The high value related to J_SC_ of 22 mA cm^−2^ is corroborating well with the incident photon-to-electron conversion efficiency (Fig. 7a) integrated J_SC_, which also confirms the high transmittance of the SnO_2_ ETL-layer.

Another advantage of the SnO_2_ as ETL for perovskite solar cells compared to TiO_2_, is its longer operational stability and lifetime. Indeed, the operational lifetime of the solar cell based on SnO_2_ ETL has been measured under its maximum power point, under a continuous light illumination, at 45 °C (the detailed results are not shown here but will be a subject for a separate report). The best-reached T80 lifetime was 250 h with the average lifetime over 200 h. This protocol is also regarded to be the most reliable and reproducible way to test the operational stability^56^.

**Figure 8:**
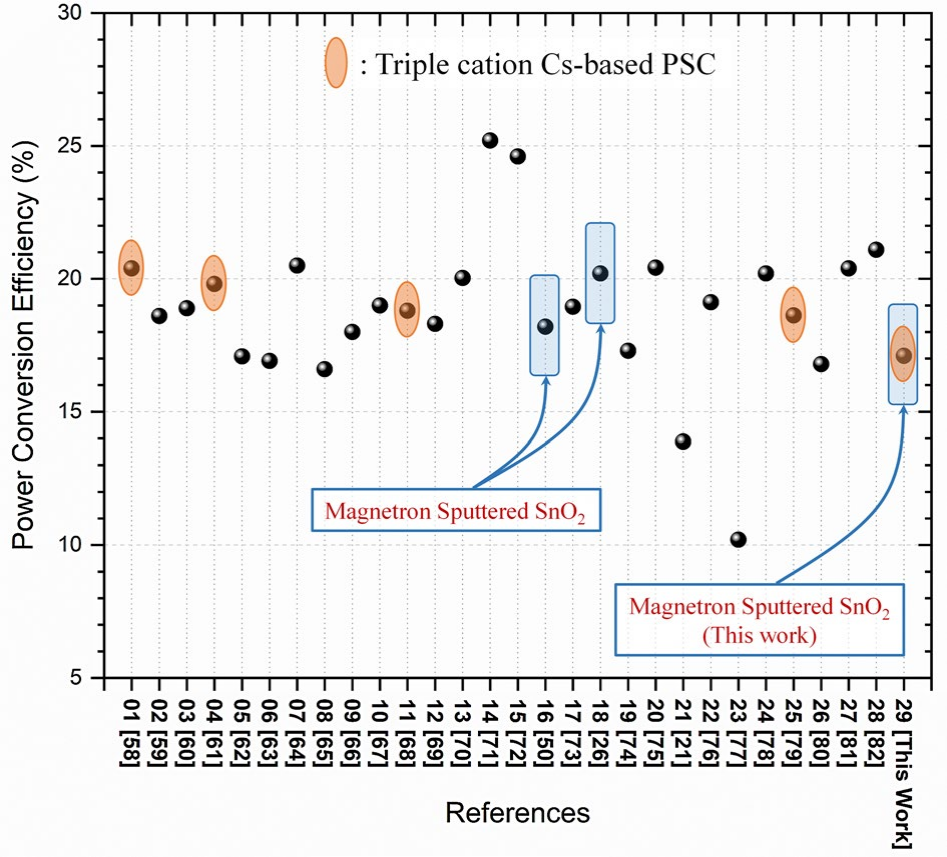
Summary of a literature survey of various PCE values recorded for different PSC solar cells based SnO_2_ ETL. SnO_2_ was grown by different methods from different sources^21,26,49,57–81^.

Finally, Fig. 8 summarizes a literature survey of various PCE values recorded for different PSC solar cells based SnO_2_ ETL from about thirty references. In this literature survey, SnO_2_ ETL was grown by different methods from different material sources. Only two references related to magnetron sputtering were found and show values comparable to our current work. Further optimization might be provided to enhance the PV properties through a systematic study by improving the microstructural and optoelectronic properties via post-deposition thermal annealing.

## Conclusions

We have successfully employed the RF magnetron sputtered SnO_2_ as ETL for spin-coated-(FA, MA, and Cs) triple cation-based perovskite solar cells on FTO substrates. We demonstrated that neither mesoporous scaffold nor high-temperature processing procedures were essential to achieve high device performance. In addition, no passivation process has been performed and no encapsulation has been used. Nevertheless, PSCs of 17.10% PCE have been achieved. Material’s characterization study has demonstrated that the air annealing has enhanced the material’s structural and electrical properties, particularly the improvement in crystallite size which led to an improvement in electron mobility. These enhancements have improved the overall performance of the PSC device. Our results on the temperature dependency suggested an interplay of different physical phenomena, including charge transfer dynamics and charge recombination that govern the PV performance at different temperature intervals. We attributed the PL changes up to 40 °C to the possible carrier accumulation near the SnO_2_/perovskite interface and to the diminution in the non-radiative charge traps, whereas higher charge–phonon interactions are believed to be dominating at higher temperatures (i.e. from 40 to 75 °C). Our work offers a significant insight into the operational reliability and variability of the PV performance of PSCs, as well as the underlying mechanisms in perovskite materials, at real-world operating temperatures. More importantly, sputtered SnO_2_ as ETL was demonstrated to enable a good PV device performance, stability and lifetime and could serve as a promising route for further development and integration of sputtered SnO_2_ films into large-scale and cost-effective perovskite PV modules.

## Methods

### Electron transport layer (ETL)

The glass coated FTO substrates with 7Ω/□ sheet resistance were first cleaned with 1 wt% Na dodecylsulfate aqueous, deionized water, acetone, and isopropyl alcohol prior to their utilization. SnO_2_ thin films were grown on these Glass/FTO by RF magnetron sputtering (Torr™) at 200 °C, of a high purity SnO target (purity 99.99%, 2″ diameter, Codex International™), under O_2_:Ar mixture at 4:200 sccm and a power of 50 W for 10 min. A base pressure of 5 × 10^–5^ Torr was first reached and then a deposition pressure which depends on the variable oxygen flow rate, was set around 5 × 10^–3^ Torr. A sufficient oxygen flow was provided to assure the highest chemical state of Sn oxide in the grown film which are Sn(IV) oxide, while argon is necessary to form and maintain the magnetron plasma. The film thickness was about 40 nm. A deposition rate of about 4 nm min^−1^ was deliberately chosen for a better film’ coverage. Films were then treated with UV-ozone for 15 min and then one SnO_2_ sample was post annealed at 250 °C in air, for 30 min. Samples were then placed into a nitrogen-filled glovebox for the perovskite layer deposition.

### Perovskite absorber layer

“The (FA, MA, and Cs: FMC) triple-cation mixed-halide perovskite was prepared inside glovebox conditions to maintain oxygen and H_2_O levels under 1 ppm. The one-step and ‘antisolvent’ processes were employed to obtain FAPbI3 and FMC based films. Cs_0.05_MA_0.10_FA_0.85_Pb(I_0.85_Br_0.15_)_3_ was prepared by dissolving the precursors in a 1:4 DMSO: DMF mixed solvent. A 1.4 M precursor solution was obtained by mixing CsI (0.07 M), FAI (1.13 M), PbI_2_ (1.19 M), MABr (0.2 M), and PbBr_2_ (0.2 M) in the DMSO: DMF solvent with a 5% excess of PbI2. The perovskite films were deposited onto the 40-nm SnO_2_ layer by spin-coating. This process started at 1000 rpm, for 10 s with a first-step acceleration rate of 200 rpm/s, followed by a second step acceleration at 1500 rpm/s until reaching a final speed of 4000 rpm, and then maintained for 35 s. During the last 20 s of spinning, 120 μL of chlorobenzene was dropped onto the substrate. The obtained film was finally annealed at 100 °C for 60 min”, this process is reported previously by Manekkathodi et al.^82^.

### Hole-transport layer (HTL)

“The doped Spiro-OMeTAD was prepared by dissolving 65 mg of Spiro-OMeTAD in 1 mL of chlorobenzene. Then, 17.5 μL/mL of 26 mM solution of Li-dopant (bis(trifluoromethane)-sulfonimide lithium salt solution in acetonitrile) in acetonitrile, 21.9 μL/mL of 26 mM solution of cobalt-dopant (FK209) solution in acetonitrile, and 20 μL/mL of tert-butylpyridine were added as an additive. This solution was spin-coated at 4000 rpm for 20 s on the perovskite films immediately after their growth. After the Spiro-OMeTAD deposition, samples were taken out from the glovebox for oxidation purposes”, this process is also reported previously by Manekkathodi et al.^82^.

### Back-contact electrode

Back-contact electrode of 100 nm Au film was thermally evaporated under vacuum to complete the device. Film thicknesses were measured by a stylus profiler (Bruker Dektak).

### Optical properties

UV and visible light Transmittance and Absorbance spectra were determined by UV–visible spectrometry (Jasco V670).

### Electrical properties

Electrical properties were probed through Hall effect measurements using a van der Pauw measurement technique. A probe-Lakeshore 8400 equipment, with a magnetic field of 0.56 T was used. Measurements were performed at room temperature, in air, and at atmospheric pressure. The electrical properties (including carrier mobility) were determined through the Hall voltage by forcing both a magnetic field perpendicular to the sample (i.e. the film) and a current through the sample. The combination of the current flow and the magnetic field causes a transverse current. The resulting potential is measured across the film.

### Solar cell device performance

A standard AM1.5G AAA solar simulator with 100 mW/cm^2^ and a certified reference Si solar cell was employed to analyze the PV performance (Newport, Inc.). Measurements were performed in ambient air. An appropriate mask designed an active area of 0.16 cm^2^. I–V plots were recorded under different temperatures between 25 and 75 °C upon illumination (Keithley Model 2400 source meter). The temperature ramp/cooling rate of 3–5 °C/min was adopted through a solid-state heating/cooling system (ThermoCube). The PSCs were stabilized at each temperature setting for 10 min prior to launch the subsequent measurement. All the cooling and/or heating steps were performed under dark conditions.

A load resistance of 180 Ω was connected to the device for its stability. Current–voltage (I–V curve) scans were acquired. No filters have been used during the measurements. The stability measurement was performed at RH of 5% in a nitrogen box. The EQE scans were acquired by an IPCE measurement system.

### Temperature-dependent PL

An iHR320 fluorescence spectrometer (Horiba, Jobin Yvon iHR320) equipped with CCD camera was used for the photoluminescence (PL) spectra recording. To decouple the response of the absorbing material from any other influence, only perovskite films were grown on glass substrate following the same procedure depicted above. A green PL excitation source of 532 nm wavelength was used and based on a diode-pumped solid-state laser (from Laser Quantum).

The aim is to probe the temperature dependency of the PL response of the absorber films. To do so, the temperature was varied from RT to 75 °C with a ramping rate of 5 °C/min. At each temperature, we stabilize the sample’s temperature for 5 min prior to its measurement. Laser was turned off during the heating and stabilization intervals to avoid any photo-degradation of the samples. For all the temperatures, the PL response was recorded from the same spot on the sample and with the same exposure and integration-time.

### PSC device configuration and cross-sectional imaging

**Figure 9:**
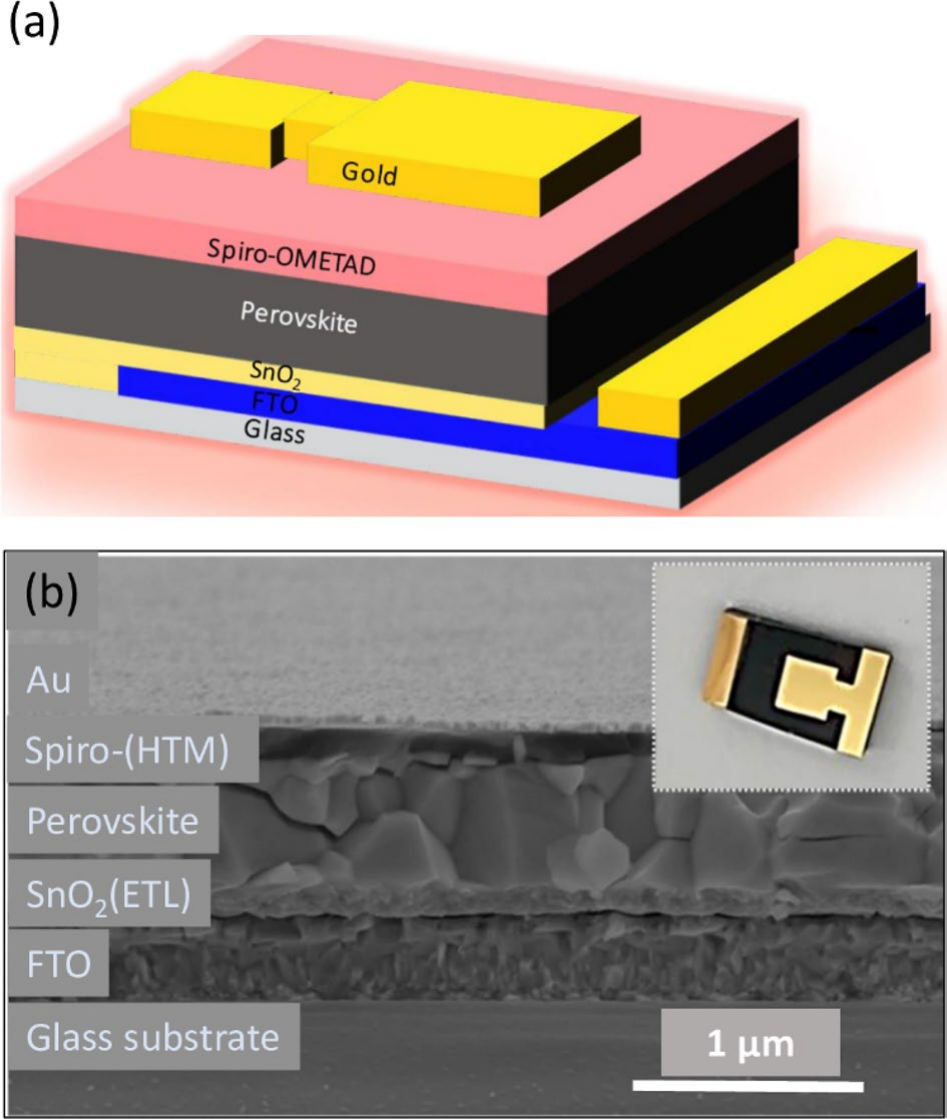
(**a**) Schematic diagram of the perovskite solar cell (PSC) used in this study. (**b**) Cross-sectional SEM image of Cs_0.05_MA_0.10_FA_0.85_Pb(I_0.85_Br_0.15_)_3_ PSC. The inset shows a photograph of a PSC that is representative of those used in our experiments.

The PSC device configuration is based on the conventional architecture where the layers on the glass substrate are fluorine doped tin oxide (FTO), SnO_2_, perovskite, Spiro-OMETAD and Au, respectively as shown in Fig. 9. FTO material and SnO_2_ are used as anode and ETL, respectively, while the perovskite as the absorber layer. Spiro-OMETAD acts as HTL, and the metallic cathode is made of Au contact.

### Electron transport layer (ETL) characterization

SnO_2_ samples were prepared on glass substrates to characterize solely the SnO_2_ materials properties without the influence of FTO.

*Grazing incidence X-ray diffraction (GIXRD)* has been performed for both as-deposited SnO_2_ and the annealed samples. The incident angle of the X-ray source was fixed at 0.55° while the 2θ was scanned from 15° to 65° for both diffractograms. The step size was fixed at 0.02° and the scan speed was kept at 2°/min for both diffractograms. The X-ray source is Cu K-alpha, and its wavelength is 1.54 Å. GIXRD analysis was performed using Rigaku Smartlab (Japan).

*X-ray photoelectron spectroscopy (XPS)* has been conducted on both as-deposited and annealed SnO_2_ thin film samples. High resolution spectra for Sn3d and O1s were performed using 20 eV pass energy, 0.1 eV Step size and 5 periods (average of 5 spectra) while survey spectra were achieved using 100 eV pass energy, 1 eV step size and 1 period (no averaging). Prior to the XPS analysis, both samples were first cleaned using low energy/atom argon cluster source to remove the surface contamination without altering the surface chemistry of the SnO_2_ thin film samples, which is confirmed by reducing drastically the carbon contamination while keeping the Sn3d spectra intact. It is worth noting that XPS equipment is calibrated using triple pure standard samples of Au, Ag and Cu. During the measurements, all spectra were referenced using C1s to correct all the surface charging related shift. XPS analysis was performed using Thermo Fisher Scientific-Escalab 250Xi (United Kingdom).

## Data Availability

The datasets used and/or analyzed during the current study are available from the corresponding author on reasonable request.
